# Evaluation of 7 techniques for the quantification of myocardial oedema in STEMI

**DOI:** 10.1186/1532-429X-15-S1-P188

**Published:** 2013-01-30

**Authors:** Elisa McAlindon, Chris Lawton, Andrew Flett, Nathan Manghat, Mark C Hamilton, Chiara Bucciarelli-Ducci

**Affiliations:** 1 NIHR Bristol Cardiovascular Research Unit, Bristol, UK; 2 The Heart Hospital, London, UK

## Background

Myocardial oedema is a consequence of injury during ST segment elevation myocardial infarction (STEMI). T2 weighted short tau inversion recovery (T2w STIR) is widely used for oedema assessment on CMR. No consensus exits for which threshold/ contouring method should be used for the quantification of the area of myocardial oedema as a surrogate endpoint in clinical trials. This study investigates the inter- and intra- observer and inter-scan reproducibility of 7 techniques in use for oedema assessment (2 SD, 3 SD, 5 SD, FWHM, Otsu, manual threshold and manual contouring). The aim was to determine the most robust method for the quantification of myocardial oedema STEMI.

## Methods

20 patients day 2 following acute reperfused STEMI were assessed. All patients had 2 CMR scans on the same day at least 6 hours apart. These CMRs included a full short axis stack (8mm slice thickness) of T2w STIR. Images were analysed offline using CMR 42 software (Circle CVI). The endocardium and epicardium were delineated on each slice. For 2, 3 and 5 SD a region of interest was drawn in the remote myocardium, deemed unaffected with the absence of RWMA or LGE. In addition a ROI was drawn in the high signal intensity myocardium in the affected myocardium for the FWHM technique. Manual thresholding was subjective as was manual contouring. The myocardial oedema was expressed as a % of LV. The difference between techniques was assessed using a 1 way ANOVA. The inter-, intra-observer and inter-scan agreement was assessed using the Bland Altman method. Variability was calculated as 1- intraclass correlation coefficient (ICC).

## Results

There is a significant difference in the % of LV with myocardial oedema between all 7 techniques used (p<0.001). 5SD produced the smallest volume of myocardial oedema (8.7 % LV +/- 6.6), FWHM the largest (59.8 % LV +/- 15.9). Manual contouring provided the best inter-, intra- observer and best inter-scan agreement using Bland Altman, with lowest variability (Figure 2). Manual thresholding had the worst agreement.

**Figure 1:**
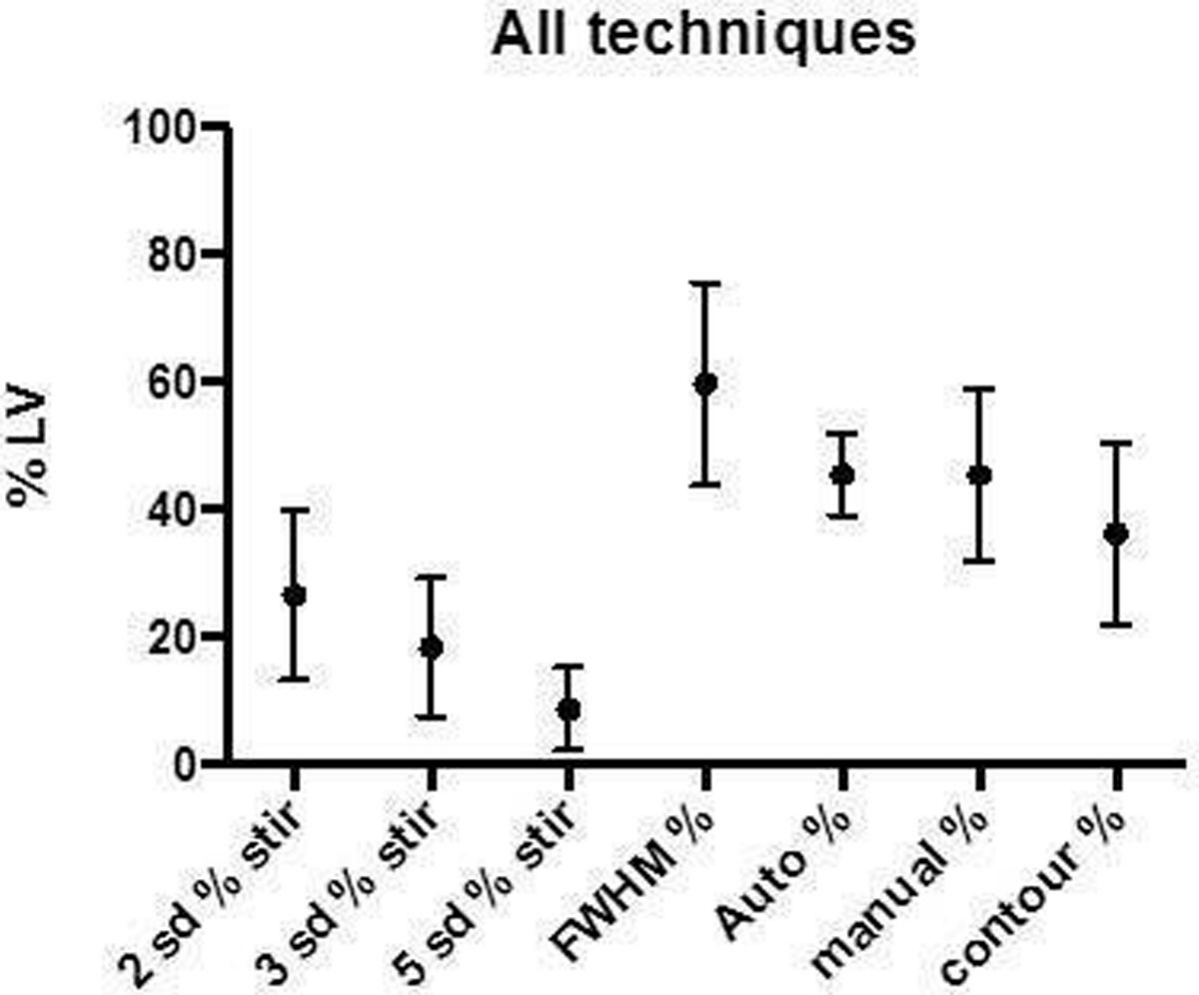
Quantification of myocardial oedema by 7 different techniques.

**Figure 2:**
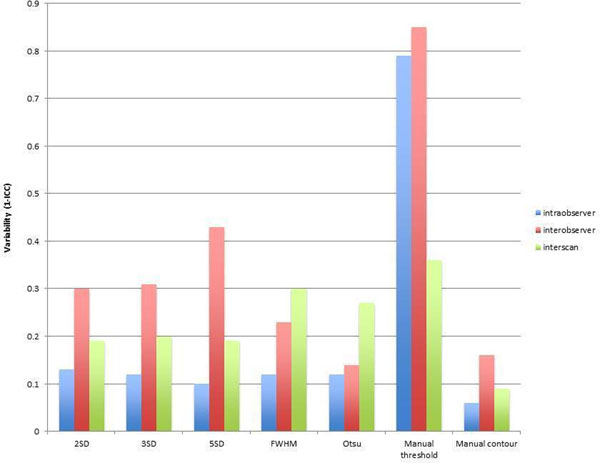
Intra- and inter-observer and interscan variability for the different techniques to quantify oedema (1- intraclass correlation coefficient(ICC)).

## Conclusions

This study supports the manual contouring method as the most robust for quantification of myocardial oedema as a surrogate endpoint in clinical trials. The Otsu method provided good agreement between observers and scans and is a robust method. Manual thresholding should not be used.

## Funding

This study was funded by the NIHR Bristol Cardiovascular Biomedical Research Unit.

